# Association between post-transplant serum uric acid levels and kidney transplantation outcomes

**DOI:** 10.1371/journal.pone.0209156

**Published:** 2018-12-14

**Authors:** Deok Gie Kim, Hoon Young Choi, Ha Yan Kim, Eun Ju Lee, Kyu Ha Huh, Myoung Soo Kim, Chung Mo Nam, Beom Seok Kim, Yu Seun Kim

**Affiliations:** 1 Department of Transplantation Surgery, Severance Hospital, Yonsei University Health System, Seoul, South Korea; 2 Department of Internal Medicine, Yonsei University College of Medicine, Seoul, South Korea; 3 Biostatistics Collaboration Unit, Yonsei University College of Medicine, Seoul, South Korea; 4 Department of Surgery, Yonsei University College of Medicine, Seoul, South Korea; 5 The Research Institute for Transplantation, Yonsei University College of Medicine, Seoul, South Korea; 6 Department of Preventive Medicine, Yonsei University College of Medicine, Seoul, South Korea; University of Cambridge, UNITED KINGDOM

## Abstract

**Background:**

Serum uric acid (UA) level has been reported to be associated with chronic allograft nephropathy and graft failure in patients who undergo kidney transplantation (KT). However, the role of serum UA level in renal graft survival remains controversial.

**Objective:**

This study aimed to investigate the effect of mean serum UA level during two different post-KT periods on long-term renal graft outcomes in a large population cohort in which living donor KT prevails.

**Material and methods:**

A retrospective cohort study was performed using KT data prospectively collected at a single institution. Patients (n = 2,993) were divided into low-, normal-, and high-UA groups according to the mean serum UA level within the first year (1-YR) and 1–5 years (5-YR) after transplantation.

**Results:**

In the 1-YR Cox proportional hazards analysis, the low- and high-UA groups had a significantly decreased and increased risk, respectively, for overall graft failure (OGF), death-censored graft failure (DCGF), and composite event (return to dialysis, retransplantation, death from graft dysfunction, and 40% decline in estimated glomerular filtration rate) compared with the normal-UA group. Similarly, in the 5-YR analysis, the low-UA group had a significantly reduced risk of DCGF compared with the normal-UA group, whereas the high-UA group had a significantly increased risk of all three graft outcomes. In a marginal structural model, hyperuricemia had a significant causal effect on worsening graft outcomes, with consideration of all confounding variables (OGF: hazard ratio [HR] 2.27, 95% confidence interval [CI] 1.33–3.78; DCGF: HR 2.38, 95% CI 1.09–4.9; composite event: HR 3.05, 95% CI 1.64–5.49).

**Conclusions:**

A low-to-normal serum UA level within the first year and 1–5 years after KT is an independent factor for better renal allograft outcomes in the long-term follow-up period rather than high serum UA level.

## Introduction

Kidney transplantation (KT) has been considered the best treatment for patients with end-stage renal disease. However, the exact mechanisms of renal graft failure remain unclear in pediatric and adult patients despite various studies on improvement in graft survival [1,2]. Therefore, many researchers have performed investigations to identify pathological mechanisms and risk factors for renal graft failure [3].

The mean serum uric acid (UA) level during the first 6 months after transplantation has been reported to be an independent predictor of long-term graft survival and short-term graft function [4], and early-onset hyperuricemia at 3 months after KT showed an increased risk for graft failure in the propensity-score matching analysis of a multicenter cohort study [5]. In contrast, previous randomized controlled trials have reported that serum UA level is not an independent risk factor for graft failure [5,6]. There was no association between renal function decline at 1 or 3 years and high UA levels at 1 month after KT after correcting for baseline renal function in a subanalysis of the Symphony study [6]. Furthermore, Kim et al. showed that serum UA level is not an independent risk factor for graft failure after accounting for graft function as a time-varying confounder [7].

However, numerous studies on patients with chronic kidney disease (CKD) have suggested a link between serum UA levels and renal dysfunction [8,9]. Moreover, treatment of asymptomatic hyperuricemia leads to improved patient and graft survival. Considering the link between UA level and risk of diabetes, metabolic syndrome, hypertension, and cardiovascular diseases, lowering the UA level to minimize these risk factors may be beneficial for graft function. Minimizing the use of diuretics and cyclosporine and avoiding purine-rich foods and alcohol are also effective strategies to decrease the serum UA level in KT recipients [10].

The present study aimed to investigate the effect of low, normal, or high post-transplant serum UA levels during two different post-KT periods on long-term renal graft outcomes in a large Korean population cohort in which living donor KT prevails. We used an approach that simultaneously accounts for time-varying exposures and confounders, allowing valid inferences to be made from complex longitudinal data in observational cohort studies [7,11].

## Materials and methods

### Data source

A retrospective cohort study was conducted using data of 2,993 patients who underwent KT from January 1992 to December 2014 at Severance Hospital, Yonsei University College of Medicine. The serum UA level was measured at 1, 3, 6, 9, and 12 months after transplantation and then annually throughout the study period. The serum creatinine level was also measured at the same time points, and the estimated glomerular filtration rate (eGFR) was calculated using the Chronic Kidney Disease Epidemiology Collaboration formula [12].

### Study population selection process

**Fig 1:**
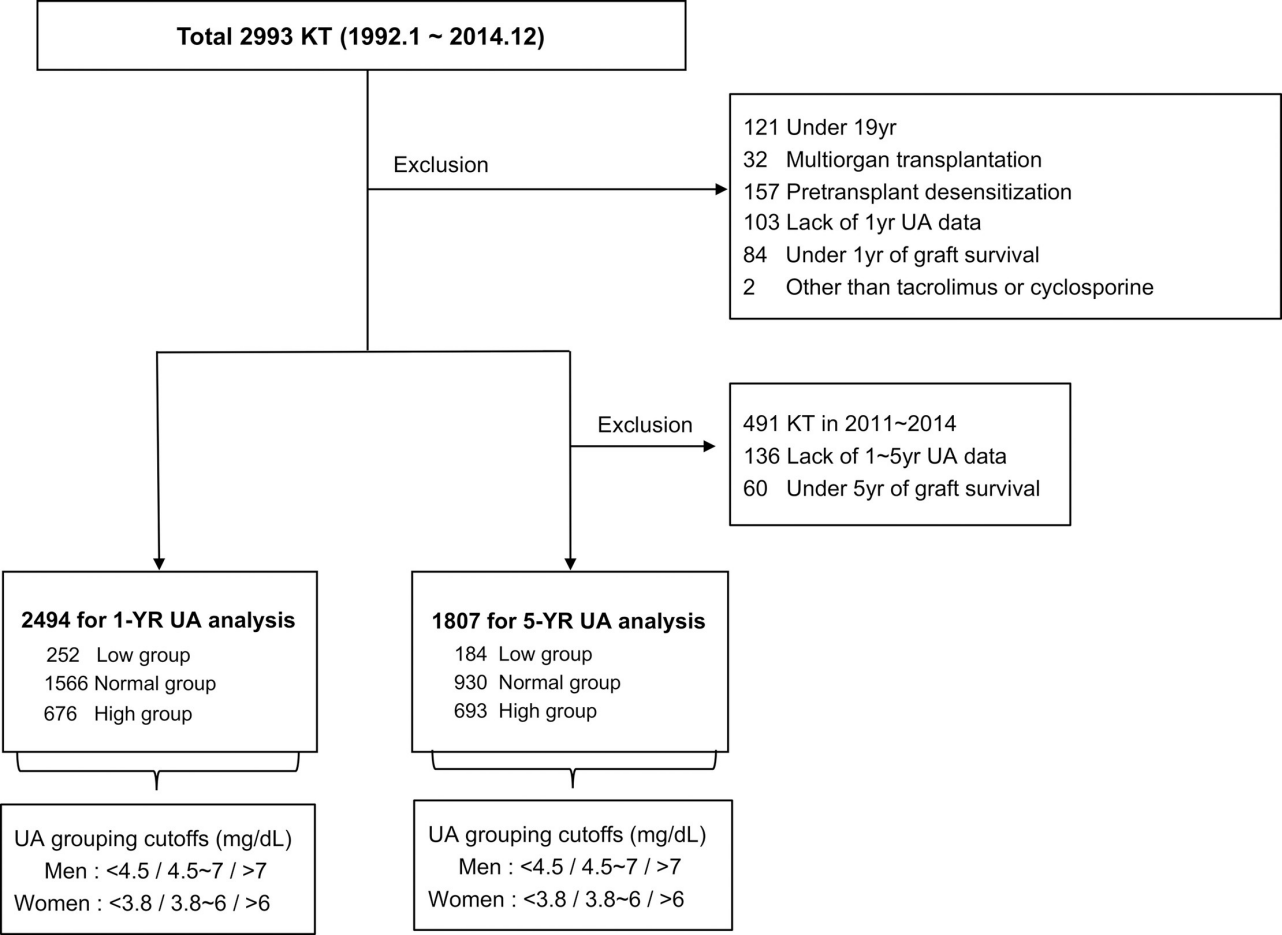
Algorithm used to define the study cohort. Uric acid (UA) grouping was performed according to the mean serum UA level within the first year (measured at 1, 3, 6, 9, and 12 months) and 1–5 years (measured annually) after transplantation in the 1-YR and 5-YR analyses, respectively.

The study population selection process and exclusion criteria are shown in Fig 1.

Patients were divided into low-UA, normal-UA, and high-UA groups according to the mean serum UA level within the first year (1-YR) and 1–5 years (5-YR) after KT. High serum UA level was defined as a mean serum UA level >7.0 mg/dL in men and >6.0 mg/dL in women. Cutoff values for the low-UA group were defined using the sex-specific 10^th^ percentile value from our data distribution (S1 Fig). Each group showed a similar serum UA level trajectory over time in the two analyses, maintaining the original grouping (S2 Fig).

Patients with grafts and survival exceeding 1 year were included (n = 2,494) in the 1-YR analysis, with a mean follow-up period of 130.9±74.4 months (maximum, 302 months), whereas patients who underwent KT from 2011 to 2014 and those with survival <5 years were excluded in the 5-YR analysis. Thus, a total of 1,807 patients were eligible for the 5-YR analysis, with a mean follow-up period of 160±64.6 months (maximum, 302 months).

The primary endpoints were overall graft failure (OGF), death-censored graft failure (DCGF), and composite event, defined as the sum of return to dialysis, retransplantation, death from graft dysfunction, and >40% decline in eGFR from the baseline level, which was measured at 1 year (1-YR analysis) or 5 years (5-YR analysis) after transplantation. The secondary endpoint was eGFR decline.

The study was performed in accordance with the Declaration of Helsinki principles and approved by the independent institutional review board of Yonsei University College of Medicine (IRB no. 4-2017-0834). Moreover, the clinical and research activities being reported are consistent with the Principles of the Declaration of Istanbul as outlined in the “Declaration of Istanbul on Organ Trafficking and Transplant Tourism.”

### Statistical analysis

Group differences in baseline characteristics were evaluated using Pearson’s chi-squared test for categorical variables and one-way analysis of variance with post-hoc testing using Bonferroni’s method for continuous variables. The association between serum UA level and graft outcomes was evaluated using Kaplan–Meier survival curves and log-rank test (low- and high-UA groups versus normal-UA group). To determine whether UA group was an independent risk factor for the three graft outcomes, Cox proportional hazards analyses were performed with the following models: 1) model 1 adjusted for transplant era, age, sex, body mass index, donor type, donor age, donor sex, number of human leukocyte antigen mismatches, pre-transplant diabetes mellitus, duration of pre-transplant dialysis, retransplantation, tacrolimus use, delayed graft function, biopsy-proven acute rejection within 1 year, systolic/diastolic blood pressure, and eGFR at 1 month after KT and 2) model 2 adjusted for covariates in model 1 with eGFR at 1 year after KT rather than at 1 month. The results of the Cox analysis are presented as hazard ratios (HRs) with 95% confidence intervals.

Repeated-measures variables, such as serum UA level and eGFR, were evaluated using a linear mixed model. We estimated eGFR variation based on the baseline value and evaluated group differences in eGFR changes over time. Considering serum UA level as a time-varying factor, time-varying Cox models were fitted with adjustment for baseline covariates to confirm the effects of serum UA level on graft outcomes. However, this analysis could not confirm a causal relationship between serum UA level and graft outcomes, as serum UA and eGFR affect each other. Therefore, we used marginal structural models (MSMs) [13] to estimate the causal effect of the time-varying serum UA level on graft outcomes in the presence of the time-varying confounder, eGFR, which is affected by the prior serum UA level. These models can be used to create a pseudo-population by implementing inverse probability of treatment weighting, which renders the relationship between serum UA level and other confounders, especially eGFR, independent. Because small errors could result in large weights, it was difficult to estimate the density of the serum UA level as a continuous or polytomous variable. Therefore, only two UA categories (high and normal) were used in the time-varying Cox models and MSMs. Multiple imputation using chained equations was used to handle missing data. Analyses were performed on five sets of imputed data, and estimates were combined using Rubin’s rules.

Most statistical analyses were performed using SPSS version 23.0 (IBM Corp., Armonk, NY, USA). SAS version 9.4 (SAS Institute Inc., Cary, NC, USA) was used for the linear mixed model and time-varying Cox analyses, whereas R software version 3.4.3 (R Foundation for Statistical Computing, Vienna, Austria) was used for the MSMs and multiple imputation. *P*-values <0.05 were considered significant.

## Results

### Baseline characteristics

The baseline characteristics are summarized in Tables 1 and 2 for the 1-YR and 5-YR analyses, respectively.

**Table 1:** Comparisons of baseline characteristics among UA groups according to the mean serum UA level within the first year after transplantation in the 1-YR analysis.

	UA groups for 1-YR analysis			
Variables	Low (*n* = 252)	Normal (*n* = 1566)	High (*n* = 676)	*P* value
Transplant era				<0.001
1992–2003	94 (37.3%)†‡	751 (47.8%)*‡	426 (62.6%)*†	
2004–2014	158 (62.7%)	819 (52.2%)	254 (37.4%)	
Age (years)	43.5 ± 11.3†‡	41.3 ± 11.1*‡	39.5 ± 10.9*†	<0.001
Sex, male	158 (62.7%)	999 (63.6%)	430 (63.2%)	0.95
BMI (kg/m^2^)	21.7 ± 2.9	21.8 ± 3.1	22.0 ± 3.2	0.12
Donor type, deceased	15 (6.0%)†‡	211 (13.4%)*‡	146 (21.5%)*†	<0.001
Donor age (years)	38.0 ± 10.2‡	39.0 ± 11.9	40.1 ± 12.5*	0.02
Donor sex, male	108(42.9%)†‡	870 (55.4%)*	400 (58.8%)*	<0.001
Pretransplant DM	47 (18.7%)†‡	169 (10.8%)*‡	23 (3.4%)*†	<0.001
Duration of pretransplant dialysis (months)	15.4 ± 29.4†‡	26.4 ± 40.2*‡	36.3 ± 46.2*†	<0.001
Retransplantation	14 (5.6%)	143 (9.1%)	64 (9.4%)	0.15
Number of HLA mismatches	2.5 ± 1.3	2.5 ± 1.2	2.4 ± 1.2	0.60
Tacrolimus use	101 (40.1%)†	624 (39.7%)*	236 (34.7%)	0.07
Delayed graft function	1 (0.4%)†‡	49 (3.1%)*‡	53 (7.8%)*†	<0.001
BPAR within 1 year	17 (6.7%)†‡	295 (18.8%)*‡	225 (33.1%)*†	<0.001
SBP at 1 month (mmHg)	129.3 ± 13.7‡	129.9 ± 14.4‡	132.8 ±14.6*†	<0.001
DBP at 1 month (mmHg)	83.0 ± 10.2‡	83.2 ± 10.3‡	85.0 ± 10.9*†	0.001
eGFR at 1 month (mL/min)	72.9 ± 17.9†‡	65.6 ± 19.7*‡	56.2 ± 20.9*†	<0.001
eGFR at 1 year (mL/min)	73.7 ± 16.0†‡	67.8 ± 16.9*‡	58.6 ± 17.4*†	<0.001
Mean serum UA within 1^st^ year (mg/dL)	3.69 ± 0.52†‡	5.47 ± 0.78*‡	7.68 ± 1.15*†	<0.001

*P<0.05 versus the low-UA group

†*P*<0.05 versus the normal-UA group

‡*P*<0.05 versus the high-UA group in the post-hoc analysis

UA, uric acid; BMI, body mass index; DM, diabetes mellitus; HLA, human leukocyte antigen; BPAR, biopsy-proven acute rejection; SBP, systolic blood pressure; DBP, diastolic blood pressure; eGFR, estimated glomerular filtration rate

**Table 2:** Comparisons of baseline characteristics among UA groups according to the mean serum UA level within 1–5 years after transplantation in the 5-YR analysis.

	UA groups for 5-YR analysis			
Variables	Low (*n* = 184)	Normal (*n* = 930)	High (*n* = 693)	*P value*
Transplant era				<0.001
1992–2000	71 (38.6%)	426 (45.8%)	410 (59.2%)	
2001–2010	113 (61.4%)	504 (54.2%)	283 (40.8%)	
Age (years)	43.9 ± 10.5†‡	40.6 ± 10.5*‡	37.7 ± 10.5*†	<0.001
Sex, male	117 (63.6%)	556 (59.8%)	484 (69.8%)	<0.001
BMI (kg/m^2^)	21.8 ± 3.0	21.6 ± 3.1	21.6 ± 2.9	0.72
Donor type, deceased	4 (2.2%)	66 (7.1%)	77 (11.1%)	<0.001
Donor age (years)	36.3 ± 10.0‡	37.7 ± 11.0‡	39.1 ± 12.2*†	0.005
Donor sex, male	98 (53.3%)	553 (57.3%)	388 (56.0%)	0.58
Pretransplant DM	30 (16.3%)	88 (9.5%)	19 (2.7%)	<0.001
Duration of pretransplant dialysis (months)	14.6 ± 25.0†‡	22.2 ± 35.3*	26.0 ± 39.7*	0.001
Retransplantation	10 (5.4%)	79 (8.5%)	77 (11.1%)	0.04
Number of HLA mismatches	2.4 ± 1.1	2.4 ± 1.1	2.4 ± 1.2	0.98
Tacrolimus use	71 (38.6%)	290 (31.2%)	161 (23.2%)	<0.001
Delayed graft function	0 (0%)‡	20 (2.0%)	21 (3.0%)*	0.03
BPAR within 1 year	27 (14.7%)	198 (21.3%)	227 (32.8%)	<0.001
SBP at 1 month (mmHg)	130.0 ± 14.7	130.0 ± 14.3‡	133.3 ± 14.4†	<0.001
DBP at 1 month (mmHg)	83.7 ± 10.6	83.5 ± 10.2‡	85.4 ± 10.3†	0.001
eGFR at 1 month (mL/min)	70.6 ± 18.7†‡	65.6 ± 19.7*‡	60.6 ± 20.3*†	<0.001
eGFR at 1 year (mL/min)	74.7 ± 17.1†‡	68.9 ± 16.0*‡	60.6 ± 16.0*†	<0.001
Mean serum UA between 1 and 5 year (mg/dL)	3.86 ± 0.62†‡	5.62 ± 0.77*‡	7.83 ± 1.11*†	<0.001

*P<0.05 versus the low-UA group

†*P*<0.05 versus the normal-UA group

‡*P*<0.05 versus the high-UA group in the post-hoc analysis

UA, uric acid; BMI, body mass index; DM, diabetes mellitus; HLA, human leukocyte antigen; BPAR, biopsy-proven acute rejection; SBP, systolic blood pressure; DBP, diastolic blood pressure; eGFR, estimated glomerular filtration rate

In the 1-YR analysis, there were significant group differences in several variables known to affect graft outcomes, including transplant era, age, donor type, donor age, pre-transplant diabetes mellitus, delayed graft function, and biopsy-proven acute rejection within 1 year [14], as well as in other baseline values, such as donor sex, duration of pre-transplant dialysis, and systolic/diastolic blood pressure at 1 month after KT. In contrast, sex, body mass index, number of human leukocyte antigen mismatches, and tacrolimus use were similar among the groups in the 1-YR analysis. In addition, the eGFR at 1 month after KT was significantly lower in the high-UA group than in the normal-UA group and in the normal-UA group than in the low-UA group. Similar group differences in variables except for sex, donor sex, retransplantation, and tacrolimus use were observed in the 5-YR analysis.

### Graft outcomes

**Fig 2:**
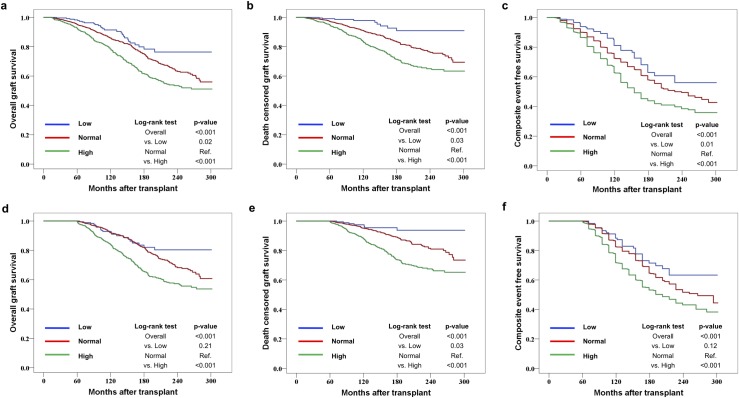
Kaplan–Meier estimates for overall graft survival, death-censored graft survival, and composite event-free survival. (a), (b), and (c) show comparisons of survival according to the mean serum uric acid (UA) level within the first year after transplantation (1-YR analysis); (d), (e), and (f) show comparisons of survival according to the mean serum UA level within 1–5 years after transplantation (5-YR analysis). The composite event represents the sum of return to dialysis, retransplantation, death from graft dysfunction, and eGFR decline of more than 40% from the baseline level, which was measured at 1 year or 5 years after transplantation (for the 1-YR and 5-YR analyses, respectively). The log-rank *P*-values for the low- and high-UA groups were estimated using the normal-UA group as reference. eGFR, estimated glomerular filtration rate.

In the 1-YR analysis, there were significant group differences in the Kaplan–Meier survival curves for overall graft survival, death-censored graft survival, and composite event-free survival. The low- and high-UA groups had significantly better and worse outcomes, respectively, than the normal-UA group. Similar results were obtained in the 5-YR analysis, with significantly worse outcomes in the high-UA group than in the normal-UA group; however, only death-censored graft survival was significantly higher in the low-UA group than in the normal-UA group (Fig 2).

**Fig 3:**
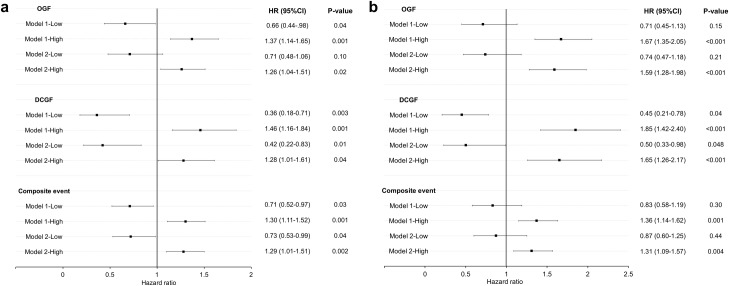
Forest plots of the Cox proportional hazards models for graft outcomes. Adjusted hazard ratio estimates and 95% confidence intervals for overall graft failure (OGF), death-censored graft failure (DCGF), and composite event are displayed according to the mean serum uric acid (UA) level within the first year (a) and 1–5 years (b) after transplantation. Covariates used in model 1 were as follows: transplant era, age, sex, body mass index, donor type, donor age, donor sex, pre-transplant diabetes mellitus, duration of pre-transplant dialysis, retransplantation, number of human leukocyte antigen mismatches, tacrolimus use, delayed graft function, biopsy-proven acute rejection within 1 year, systolic/diastolic blood pressure at 1 month, and eGFR at 1 month after kidney transplantation. Covariates used in model 2 included those used in model 1, with eGFR at 1 year after kidney transplantation rather than at 1 month. The composite event represents the sum of return to dialysis, retransplantation, death from graft dysfunction, and eGFR decline of more than 40% from the baseline level, which was measured at 1 year or 5 years after transplantation (for the 1-YR and 5-YR analyses, respectively). HR, hazard ratio; CI, confidence interval; eGFR, estimated glomerular filtration rate.

Cox proportional hazards analyses were performed to confirm that serum UA level was an independent factor affecting graft outcomes (Fig 3).

In the 1-YR analysis, the low-UA group had a lower risk of OGF, DCGF, and composite event than the normal-UA group when fitted with model 1. In model 2, the low-UA group still had a lower risk of DCGF and composite event than the normal-UA group. The high-UA group had significantly higher HRs for all three graft outcomes than the normal-UA group in models 1 and 2.

In the 5-YR analysis, the low-UA group had a significantly lower risk of DCGF than the normal-UA group in models 1 and 2. The risk of OGF and composite event did not significantly differ between the low- and normal-UA groups. The high-UA group had significantly higher HRs for all three graft outcomes than the normal-UA group in models 1 and 2. The complete results of the multivariate analyses are provided in S1–S3 Tables.

### eGFR decline

**Fig 4:**
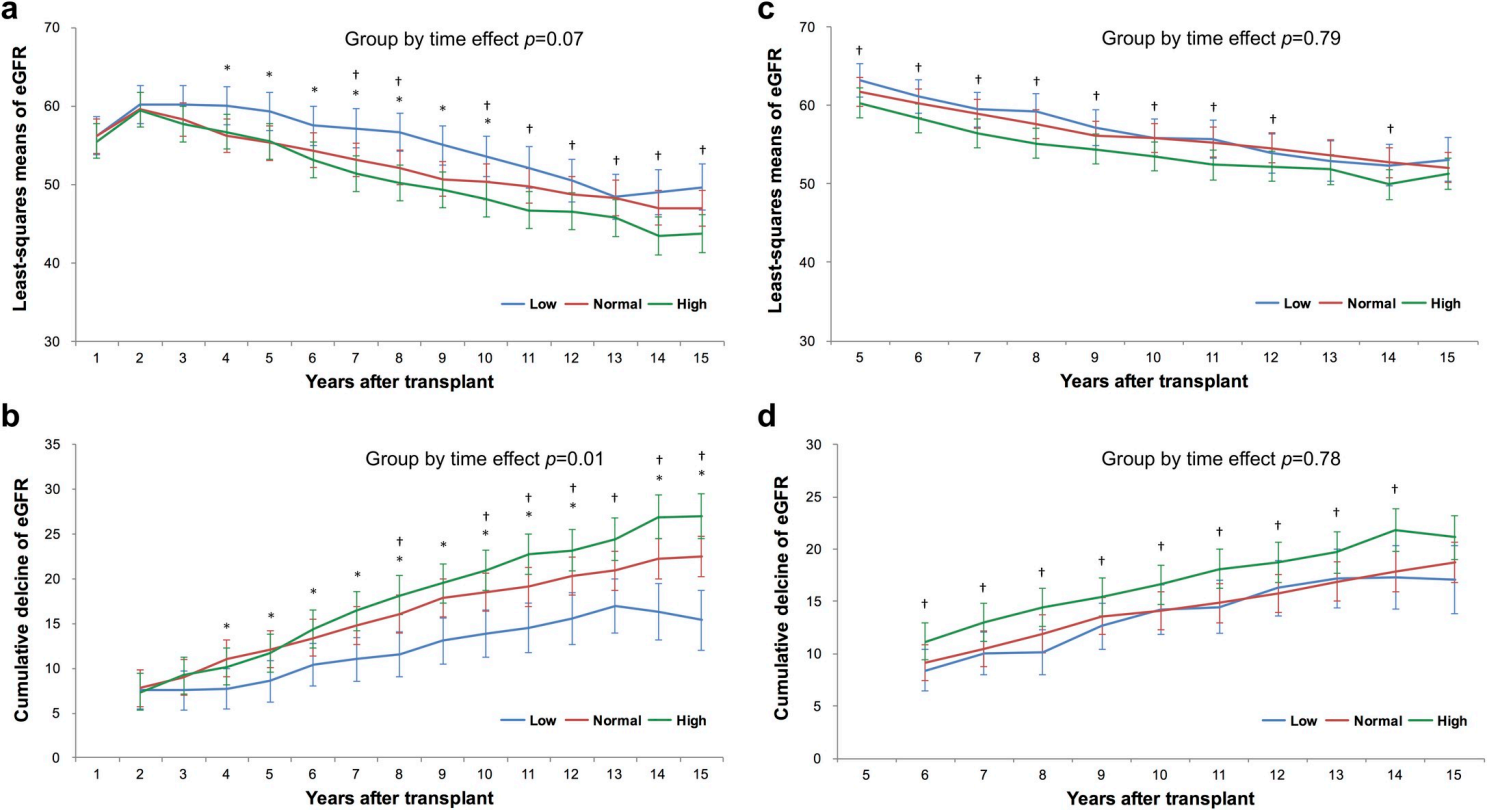
Comparisons of eGFR decline using linear mixed models. Differences in eGFR decline from the baseline level (eGFR at 1 year after transplantation) according to the mean serum uric acid (UA) level within the first year after transplantation (1-YR analysis) are shown in (a) and (b). Differences in eGFR decline from the baseline level (eGFR at 5 years after transplantation) according to the mean serum UA level within 1–5 years after transplantation (5-YR analysis) are shown in (c) and (d). The eGFR and eGFR decline are presented as least-squares means, adjusting for all covariates (including the baseline eGFR) using linear mixed models. *P*-values for the group-by-time interaction effect were calculated. *: *P*<0.05, low-UA group versus normal-UA group, †: *P*<0.05, high-UA group versus normal-UA group. eGFR, estimated glomerular filtration rate.

To evaluate the association between UA level and progression of graft dysfunction, eGFR decline was compared among the three groups (Fig 4).

Because the baseline eGFR was significantly different among groups, we used a linear mixed model to compare the least-squares (LS) mean eGFR and cumulative eGFR decline at each time point, adjusting for all confounding variables. In the 1-YR analysis, the LS mean eGFR was significantly higher in the low-UA group than in the normal-UA group, especially during the first decade. In addition, the LS mean eGFR was significantly lower in the high-UA group than in the normal-UA group, especially during later periods. Changes in the LS mean eGFR over time were not significantly different among the three groups (*P* = 0.07). However, the cumulative eGFR decline was significantly lower and higher in the low-and high-UA groups, respectively, than in the normal-UA group, mainly during later periods, with a significant group-by-time interaction effect (*P* = 0.01).

In the 5-YR analysis, the LS mean eGFR and cumulative eGFR decline were significantly lower and higher, respectively, in the high-UA group than in the normal-UA group throughout the follow-up period, whereas these measures were not significantly different between the low- and normal-UA groups. The group-by-time interactions were not significant in the 5-YR analysis (LS mean eGFR: *P* = 0.79; cumulative eGFR decline: *P* = 0.78). The results of comparisons at each time point are presented in S2 Table.

### Time-varying hyperuricemia and graft outcomes in the presence of time-varying eGFR

Hyperuricemia was significantly associated with an increased risk of all three graft outcomes in the time-varying Cox analysis, adjusting for the same covariates as those used in the conventional Cox model, plus the time-varying UA level. Furthermore, significance remained for all outcomes after imputation of missing serum UA level and eGFR values. In the MSMs, hyperuricemia still had a significant causal effect on worsening graft outcomes (OGF, DCGF, and composite event), with consideration of all confounding variables (Table 3).

**Table 3:** Association between time-varying hyperuricemia and graft outcomes.

Models	HR (95% CI)	*P value*
**Time varying Cox without imputation**^**a**^		
OGF	2.07(1.70–2.52)	<0.001
DCGF	2.08 (1.61–2.69)	<0.001
Composite event	2.73 (2.32–3.21)	<0.001
**Time varying Cox with imputation**^**b**^		
OGF	1.96 (1.59–2.43)	<0.001
DCGF	2.02 (1.55–2.63)	<0.001
Composite event	3.15 (2.6–3.81)	<0.001
**Marginal structural model**^**c**^		
OGF	2.27(1.33–3.78)	0.002
DCGF	2.38 (1.09–4.90)	0.03
Composite event	3.05 (1.64–5.49)	0.004

UA level (high or normal) was considered a time-varying dichotomous variable

^a^Adjusted for transplant era, age, sex, body mass index, donor type, donor age, donor sex, pre-transplant diabetes mellitus, duration of pre-transplant dialysis, retransplantation, number of human leukocyte antigen mismatches, tacrolimus use, delayed graft function, biopsy-proven acute rejection within 1 year, baseline systolic/diastolic blood pressure, and time-varying eGFR

^b^Multiple imputation using chained equations for the missing UA level and eGFR values

^c^Performed using the imputed dataset and the model created with consideration of all confounders, including time-varying eGFR

OGF, overall graft failure; DCGF, death-censored graft failure; HR, hazard ratio; CI, confidence interval; eGFR, estimated glomerular filtration rate; UA, uric acid

## Discussion

Average low-to-normal serum UA levels within the first year and 1–5 years after KT had a positive effect on long-term graft outcomes in KT recipients in this study. In particular, low serum UA levels after KT were associated with significantly better graft outcomes than normal serum UA levels. Consistent with previous reports, the present study also showed that hyperuricemia was associated with an increased risk of long-term graft failure compared with normal serum UA levels [15]. The mean follow-up period was over 160 months, which allowed the classification of mean serum UA levels over 1–5 years and over the first year. In addition, we estimated the causal relationship between high serum UA levels and graft outcomes using MSMs, as in previous similar studies [5,7].

Various time points have been used to investigate the causal relationship between serum UA level and graft outcomes (e.g., 1, 3, 6, and 12 months after KT) [4,6,15–20]. Previous randomized controlled trials that failed to indicate an association between mean UA levels and graft outcomes such as the Symphony study used limited time points [6]. In contrast, in the present study, serum UA levels were averaged over two different periods (within the first year and 1–5 years after KT). To our knowledge, the present study is the first to investigate whether low-to-normal serum UA levels averaged over different periods after KT are associated with better long-term graft survival in KT recipients. Although some results were similar for both analyses, some differences were observed. For example, in the 1-YR analysis, the low-UA group had a lower risk of all three graft outcomes than the normal-UA group, whereas in the 5-YR analysis, the low-UA group had a significantly lower risk of only DCGF than the normal-UA group. In addition, in the 1-YR analysis, the low-UA group had significantly lower LS mean eGFR and cumulative eGFR decline than the normal-UA group during the follow-up period. However, in the 5-YR analysis, the low- and normal-UA groups did not significantly differ with respect to both eGFR variables. In addition, the high-UA group had significantly increased HRs for OGF, DCGF, and composite event within the first year and 1–5 years after KT. Unlike those of previous studies, our results suggest that serum UA levels during different periods after KT have different associations with renal allograft outcomes [5,7,15,21,22].

The association between each cutoff value for post-transplant serum UA levels and KT outcomes has not been fully established. Higher UA levels were associated with a greater eGFR decline than a UA level of <6 mg/dL in an increasing dose–response relationship [23]. Furthermore, in patients with mild-to-severe CKD, higher serum UA level was independently associated with the risk of renal failure, although with a J-shaped relationship between UA level and all-cause mortality [24]. Low and high serum UA levels may show a similar association with unfavorable renal function outcomes, as low serum UA level can result from poor protein intake, decreased daily intake of purines or nucleotides, and malnutrition, which are associated with poor outcomes in CKD [25–27]. However, as of yet, there is no commonly accepted lower limit for the serum UA level in KT recipients. Therefore, we defined the low-UA group using the sex-specific 10^th^ percentile value (<4.5 mg/dL and <3.8 mg/dL in male and female recipients, respectively). Furthermore, high serum UA level was defined in the present study as a mean serum UA level >7.0 mg/dL in men and >6.0 mg/dL in women, consistent with previous epidemiologic studies [7,28].

In this Korean cohort, only 14.9% of patients received deceased donor KT (all from brain death). We could not confirm the superiority of low UA level in deceased donor KT owing to the scarcity of patients with low UA level. However, high UA level was still significantly associated with worse outcomes in these patients. Further studies investigating the benefit of maintaining low UA level during the early post-transplant period in deceased donor KT patients would be required.

Post-transplant hyperuricemia is associated with the use of immunosuppressive regimens that include cyclosporine, decreased glomerular filtration rate, diuretic use, and obesity during different follow-up periods [15,29–32]. An elevated serum UA level is thought to be a prognostic marker for disease progression in patients with CKD and plays an important role in the development of renal insufficiency in individuals with normal renal function [15,33–35]. Furthermore, renal injuries occur in experimental models of hyperuricemia, which can be prevented if the serum UA level is maintained within the normal range using UA-lowering agents [15,36–38].

Several mechanisms for the effect of UA on renal graft failure have been proposed. Hyperuricemia may be a consequence of reduced glomerular filtration rate in the renal allograft and might contribute to glomerular hypertrophy and tubulointerstitial fibrosis itself [15,36,37]. In addition, the serum UA level is associated with changes in renal plasma flow, arterial stiffness, and endothelial dysfunction via impairment of nitric oxide generation in vascular endothelial cells [39–41]. Furthermore, these factors can result in chronic allograft nephropathy, which is a major cause of late graft loss [42–44]. Hyperuricemia might be associated with diabetes, metabolic syndrome, hypertension, and cardiovascular diseases in KT recipients who are at risk of these conditions. The present study similarly showed that hyperuricemia was associated with an increased risk of worse renal graft outcomes in a time-varying Cox model, with consideration of the confounding effect of time-varying eGFR. In contrast to a previous report [7], hyperuricemia still had a causal effect on renal graft outcomes in the MSMs, with consideration of all confounding variables in our dataset.

The present study has several limitations. First, the study used a retrospective cohort design. Second, an extremely low serum UA level could be due to comorbid conditions common in KT recipients [45]. Third, we could not exclude the possibility that our findings were affected by dietary intake. Certain dietary habits [46], meat or seafood intake, and alcohol consumption are associated with a higher prevalence of hyperuricemia [47]. Finally, the eGFR was calculated using serum creatinine, which is dependent on muscle mass, and the generation and tubular secretion of creatinine [48,49]. Low serum creatinine levels can result from muscle wasting due to comorbid conditions in KT recipients [45].

In conclusion, a low-to-normal serum UA level within the first year and 1–5 years after KT might be an independent factor for better renal allograft outcomes in the long-term follow-up period. In addition, the present study more clearly clarifies the association between serum UA levels and KT outcomes, adjusting for time-varying confounding variables. Despite the use of diverse research designs, patient populations, and statistical methods, the effect of serum UA levels on KT outcomes remains unclear. Further experimental and clinical evidence is required to determine whether serum UA level is an independent therapeutic target for KT outcomes.

## Supporting information

S1 Fig Distribution of mean serum uric acid (UA). The distribution of mean UA within the first year after transplantation is shown in (a) and (b), whereas (c) and (d) show the mean UA from 1 to 5 years after transplantation in men and women, respectively.
(TIF)

S2 Fig Uric acid (UA) changes over time. The changes in the serum UA level over time according to the mean serum UA level within the first year after transplantation are shown in (a) and (b). The changes in the serum UA level over time according to the mean serum UA level from 1 to 5 years after transplantation are shown in (c) and (d). *: *P*-values of the group-by-time effect between the low- and normal-UA groups; **: *P*-values of the group-by-time effect between the high- and normal-UA groups.
(TIF)

S1 Table Multivariate Cox proportional hazard analysis for overall graft survival. (DOCX)

S2 Table Multivariate Cox proportional hazard analysis for death-censored graft survival. (DOCX)

S3 Table Multivariate Cox proportional hazard analysis for composite event. (DOCX)
