# Supplementary figures and images for: Association between post-transplant serum uric acid levels and kidney transplantation outcomes
Source: PLoS One. 2018 Dec 14;13(12):e0209156. doi: 10.1371/journal.pone.0209156 (PMC6294369; doi:10.1371/journal.pone.0209156)

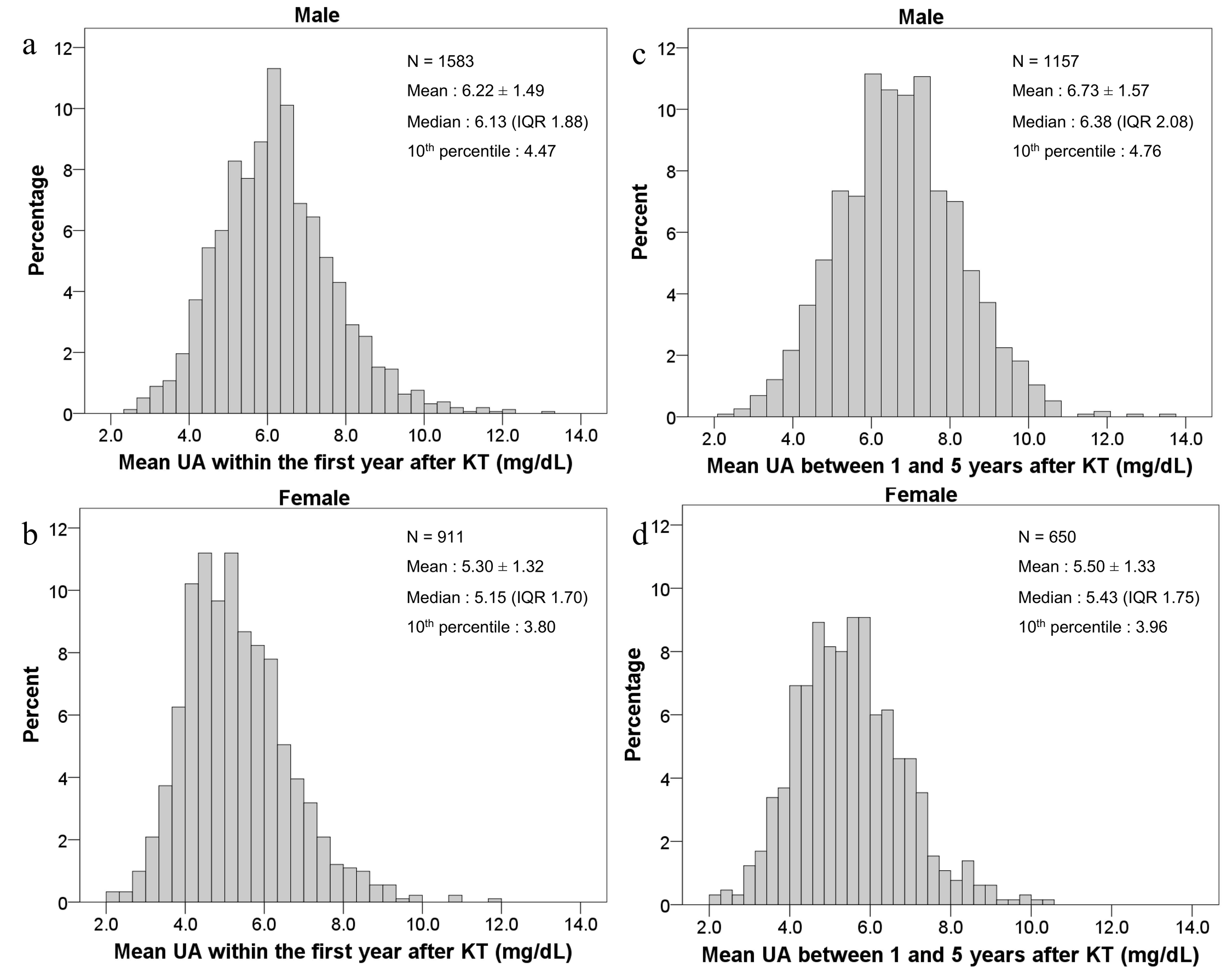

Supplement: S1 Fig — The distribution of mean UA within the first year after transplantation is shown in (a) and (b), whereas (c) and (d) show the mean UA from 1 to 5 years after transplantation in men and women, respectively. (TIF) [file pone.0209156.s001.tif]

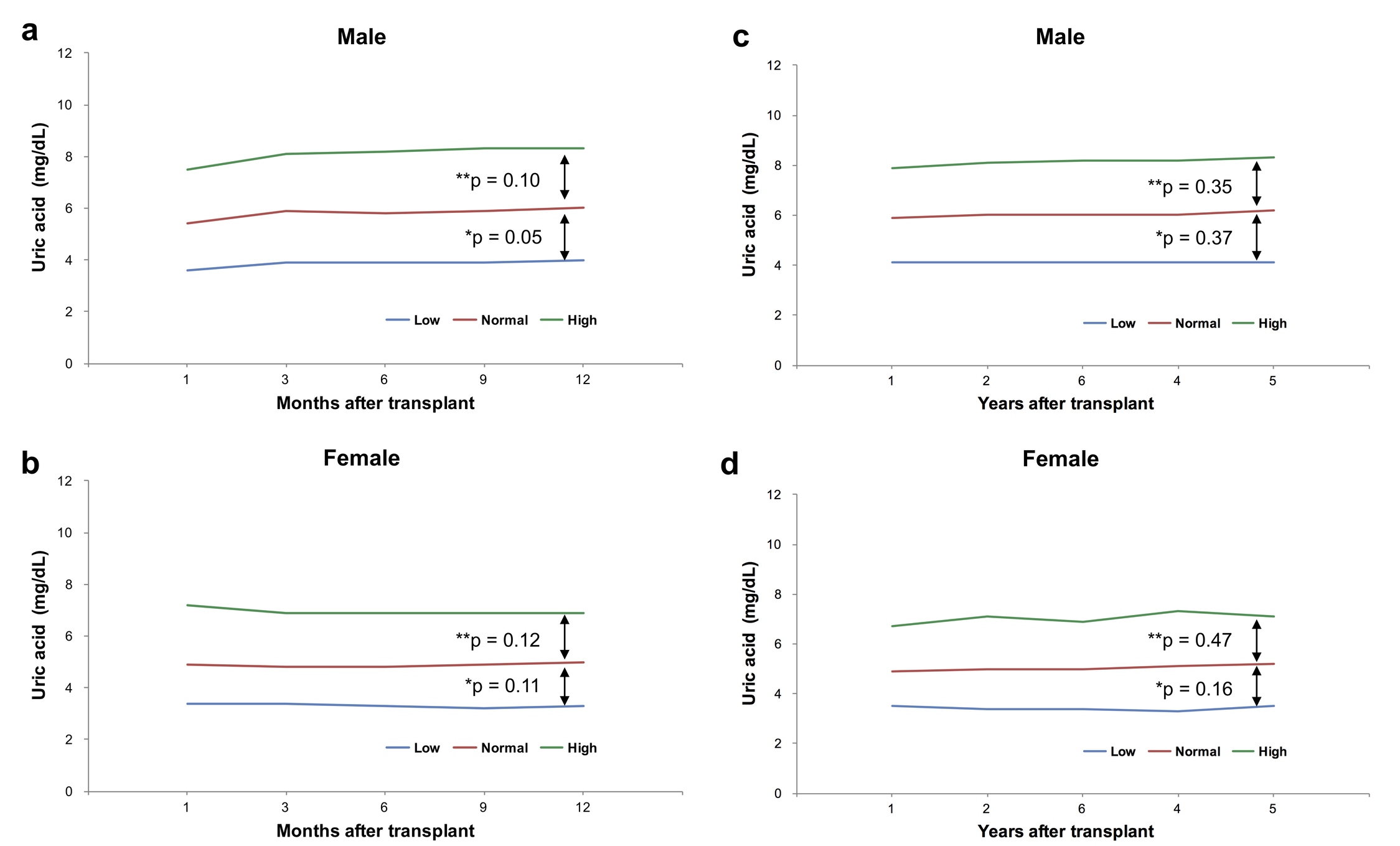

Supplement: S2 Fig — The changes in the serum UA level over time according to the mean serum UA level within the first year after transplantation are shown in (a) and (b). The changes in the serum UA level over time according to the mean serum UA level from 1 to 5 years after transplantation are shown in (c) and (d). *: P-values of the group-by-time effect between the low- and normal-UA groups; **: P-values of the group-by-time effect between the high- and normal-UA groups. (TIF) [file pone.0209156.s002.tif]
