# Supplementary material for: Association between post-transplant serum uric acid levels and kidney transplantation outcomes
Source: PLoS One. 2018 Dec 14;13(12):e0209156. doi: 10.1371/journal.pone.0209156 (PMC6294369; doi:10.1371/journal.pone.0209156)
Supplement: S1 Table — (DOCX) [file pone.0209156.s003.docx]

**Table S1. Multivariate Cox proportional hazard analysis for overall graft survival**

|  | 1-YR analysis | | | | 5-YR analysis | | | |
| --- | --- | --- | --- | --- | --- | --- | --- | --- |
|  | Model 1 | | Model 2 | | Model 1 | | Model 2 | |
| Variables | HR(95%CI) | P | HR(95%CI) | P | HR(95%CI) | P | HR(95%CI) | P |
| UA group |  |  |  |  |  |  |  |  |
| Low | 0.66 (0.44-0.98) | 0.041 | 0.71 (0.48-1.06) | 0.098 | 0.71 (0.45-1.13) | 0.150 | 0.74 (0.47-1.18) | 0.207 |
| Normal | Reference |  | Reference |  | Reference |  | Reference |  |
| High | 1.37 (1.14-1.65) | <0.001 | 1.26 (1.04-1.51) | 0.018 | 1.67 (1.35-2.05) | <0.001 | 1.59 (1.28-1.98) | <0.001 |
| Transplant era^a^ | 0.56 (0.42-0.76) | <0.001 | 0.55 (0.41-0.74) | <0.001 | 0.39 (0.29-0.54) | <0.001 | 0.38 (0.28-0.52) | <0.001 |
| Age (years) | 1.01 (1-1.02) | 0.251 | 1 (0.99-1.01) | 0.531 | 1.01 (1-1.02) | 0.239 | 1.01 (0.99-1.02) | 0.359 |
| Sex, male | 0.99 (0.83-1.2) | 0.955 | 0.96 (0.79-1.15) | 0.633 | 0.97 (0.78-1.2) | 0.763 | 0.95 (0.77-1.18) | 0.667 |
| BMI (kg/m^2^) | 1.01 (0.98-1.04) | 0.530 | 1 (0.97-1.03) | 0.860 | 1.02 (0.99-1.06) | 0.195 | 1.02 (0.99-1.06) | 0.235 |
| Donor type, deceased | 1.46 (0.99-2.15) | 0.056 | 1.43 (0.98-2.11) | 0.067 | 1.67 (1.05-2.66) | 0.030 | 1.65 (1.04-2.62) | 0.033 |
| Donor age (years) | 1.02 (1.01-1.03) | <0.001 | 1.01 (1.01-1.02) | 0.001 | 1.02 (1.01-1.03) | <0.001 | 1.01 (1-1.02) | 0.003 |
| Donor sex, male | 0.82 (0.69-0.98) | 0.030 | 0.85 (0.71-1.01) | 0.065 | 0.83 (0.67-1.01) | 0.064 | 0.83 (0.68-1.02) | 0.080 |
| Pretransplant DM | 1.94 (1.41-2.67) | <0.001 | 2.01 (1.46-2.76) | <0.001 | 2.12 (1.39-3.22) | <0.001 | 2.13 (1.4-3.24) | <0.001 |
| Duration of pretransplant dialysis (months) | 1.002 (0.999-1.004) | 0.147 | 1.002 (1-1.005) | 0.061 | 0.999 (0.996-1.003) | 0.706 | 1 (0.996-1.003) | 0.863 |
| Retransplantation | 1.17 (0.87-1.58) | 0.294 | 1.21 (0.9-1.63) | 0.202 | 1.29 (0.93-1.8) | 0.126 | 1.31 (0.94-1.82) | 0.108 |
| Number of HLA mismatch | 1.08 (1-1.17) | 0.053 | 1.09 (1-1.18) | 0.044 | 1.15 (1.05-1.27) | 0.004 | 1.16 (1.05-1.27) | 0.004 |
| Tacrolimus use | 0.85 (0.65-1.11) | 0.233 | 0.79 (0.6-1.03) | 0.083 | 0.99 (0.69-1.41) | 0.942 | 0.95 (0.66-1.35) | 0.771 |
| Delayed graft function | 1.68 (0.96-2.95) | 0.070 | 1.55 (0.89-2.71) | 0.124 | 1.45 (0.64-3.27) | 0.373 | 1.39 (0.61-3.17) | 0.427 |
| BPAR within 1 year | 1.2 (0.98-1.46) | 0.072 | 1.12 (0.92-1.35) | 0.258 | 1.04 (0.83-1.31) | 0.718 | 1.02 (0.82-1.27) | 0.861 |
| SBP at 1 month  (mmHg) | 0.996 (0.984-1.008) | 0.500 | 0.997 (0.984-1.009) | 0.576 | 0.997 (0.983-1.011) | 0.682 | 0.997 (0.983-1.012) | 0.698 |
| DBP at 1 month  (mmHg) | 1.010 (1.001-1.018 | 0.028 | 1.010 (1.001-1.018) | 0.028 | 1.006 (0.996-1.017) | 0.214 | 1.006 (0.996-1.017) | 0.213 |
| eGFR at 1month (mg/min/1.73m^2^) | 0.999 (0.994-1.004) | 0.761 |  |  | 0.999 (0.993-1.005) | 0.694 |  |  |
| eGFR at 1 year (mg/min/1.73m^2^) |  |  | 0.987 (0.981-0.993) | <0.001 |  |  | 0.994 (0.986-1.001) | 0.097 |

^a^ : after 2004 for the 1-yr analysis, after 2000 for the 5-yr analysis

UA, uric acid; BMI, body mass index; DM, diabetes mellitus; HLA, human leukocyte antigen; BPAR, biopsy-proven acute rejection; SBP, systolic blood pressure; DBP, diastolic blood pressure; eGFR, estimated glomerular filtration rate; HR hazard ratio; CI, confidence interval
