# Supplementary material for: Association between post-transplant serum uric acid levels and kidney transplantation outcomes
Source: PLoS One. 2018 Dec 14;13(12):e0209156. doi: 10.1371/journal.pone.0209156 (PMC6294369; doi:10.1371/journal.pone.0209156)
Supplement: S2 Table — (DOCX) [file pone.0209156.s004.docx]

**Table S2. Multivariate Cox proportional hazard analysis for death censored graft survival**

|  | 1-YR analysis | | | | 5-YR analysis | | | |
| --- | --- | --- | --- | --- | --- | --- | --- | --- |
|  | Model 1 | | Model 2 | | Model 1 | | Model 2 | |
| Variables | HR(95%CI) | P | HR(95%CI) | P | HR(95%CI) | P | HR(95%CI) | P |
| UA group |  |  |  |  |  |  |  |  |
| Low | 0.36(0.18-0.71) | 0.003 | 0.42(0.22-0.83) | 0.012 | 0.45 (0.21-0.99) | 0.046 | 0.50 (0.23-1.09) | 0.082 |
| Normal | Reference |  | Reference |  | Reference |  | Reference |  |
| High | 1.46(1.16-1.84) | 0.001 | 1.28(1.01-1.61) | 0.038 | 1.85 (1.42-2.40) | <0.001 | 1.65 (1.26-2.17) | <0.001 |
| Transplant era^a^ | 0.87 (0.6-1.27) | 0.480 | 0.84 (0.59-1.21) | 0.362 | 0.41 (0.28-0.61) | <0.001 | 0.38 (0.26-0.57) | <0.001 |
| Age (years) | 0.96 (0.95-0.97) | <0.001 | 0.96 (0.94-0.97) | <0.001 | 0.96 (0.94-0.97) | <0.001 | 0.95 (0.94-0.97) | <0.001 |
| Sex, male | 0.93 (0.73-1.17) | 0.527 | 0.89 (0.7-1.12) | 0.307 | 0.9 (0.68-1.18) | 0.431 | 0.88 (0.67-1.15) | 0.352 |
| BMI (kg/m^2^) | 1.02 (0.98-1.06) | 0.415 | 1.01 (0.97-1.04) | 0.794 | 1.03 (0.98-1.07) | 0.280 | 1.02 (0.98-1.07) | 0.361 |
| Donor type, deceased | 1.64 (1.01-2.67) | 0.047 | 1.55 (0.95-2.52) | 0.079 | 2.19 (1.26-3.83) | 0.006 | 2.1 (1.2-3.65) | 0.009 |
| Donor age (years) | 1.02 (1.01-1.03) | <0.001 | 1.01 (1-1.02) | 0.017 | 1.02 (1.01-1.03) | 0.001 | 1.01 (1-1.02) | 0.032 |
| Donor sex, male | 0.9 (0.72-1.12) | 0.340 | 0.95 (0.77-1.19) | 0.664 | 0.95 (0.74-1.23) | 0.713 | 0.98 (0.76-1.27) | 0.901 |
| Pretransplant DM | 2.31 (1.47-3.62) | <0.001 | 2.45 (1.56-3.86) | <0.001 | 4 (2.35-6.81) | <0.001 | 4.17 (2.44-7.11) | <0.001 |
| Duration of pretransplant dialysis (months) | 1.001 (0.997-1.004) | 0.698 | 1.002 (0.998-1.005) | 0.353 | 0.999 (0.995-1.004) | 0.720 | 1 (0.996-1.005) | 0.952 |
| Retransplantation | 1.32 (0.91-1.92) | 0.147 | 1.39 (0.96-2.02) | 0.085 | 1.36 (0.89-2.08) | 0.150 | 1.39 (0.91-2.12) | 0.126 |
| Number of HLA mismatch | 1.05 (0.95-1.17) | 0.329 | 1.06 (0.96-1.17) | 0.273 | 1.11 (0.98-1.26) | 0.087 | 1.11 (0.99-1.26) | 0.082 |
| Calcineurin inhibitor (Tacrolimus) | 0.82 (0.58-1.16) | 0.260 | 0.73 (0.52-1.03) | 0.074 | 1.14 (0.73-1.78) | 0.562 | 1.03 (0.66-1.6) | 0.897 |
| Delayed graft function | 1.7 (0.83-3.49) | 0.144 | 1.48 (0.73-3.02) | 0.282 | 1.67 (0.68-4.1) | 0.263 | 1.77 (0.72-4.37) | 0.218 |
| BPAR within 1 year | 1.37 (1.07-1.76) | 0.012 | 1.17 (0.92-1.48) | 0.206 | 1.1 (0.83-1.46) | 0.518 | 1.02 (0.78-1.33) | 0.898 |
| SBP at 1 month  (mmHg) | 1.002 (0.986-1.017) | 0.838 | 1.003 (0.988-1.018) | 0.720 | 1.002 (0.985-1.02) | 0.802 | 1.003 (0.985-1.021) | 0.775 |
| DBP at 1 month  (mmHg) | 1.010 (0.999-1.021) | 0.074 | 1.009 (0.998-1.021) | 0.091 | 1.0047 (0.994-1.02) | 0.274 | 1.007 (0.994-1.02) | 0.302 |
| eGFR at 1month (mg/min/1.73m^2^) | 1.002 (0.995-1.008) | 0.628 |  |  | 0.998 (0.991-1.005) | 0.608 |  |  |
| eGFR at 1 year (mg/min/1.73m^2^) |  |  | 0.981 (0.973-0.989) | <0.001 |  |  | 0.985 (0.976-0.995) | 0.003 |

^a^ : after 2004 for the 1-yr analysis, after 2000 for the 5-yr analysis

UA, uric acid; BMI, body mass index; DM, diabetes mellitus; HLA, human leukocyte antigen; BPAR, biopsy-proven acute rejection; SBP, systolic blood pressure; DBP, diastolic blood pressure; eGFR, estimated glomerular filtration rate; HR hazard ratio; CI, confidence interval
