# Supplementary material for: Association between post-transplant serum uric acid levels and kidney transplantation outcomes
Source: PLoS One. 2018 Dec 14;13(12):e0209156. doi: 10.1371/journal.pone.0209156 (PMC6294369; doi:10.1371/journal.pone.0209156)
Supplement: S3 Table — (DOCX) [file pone.0209156.s005.docx]

**Table S3. Multivariate Cox proportional hazard analysis for composite event.**

|  | 1-YR analysis | | | | 5-YR analysis | | | |
| --- | --- | --- | --- | --- | --- | --- | --- | --- |
|  | Model 1 | | Model 2 | | Model 1 | | Model 2 | |
| Variables | HR(95%CI) | P | HR(95%CI) | P | HR(95%CI) | P | HR(95%CI) | P |
| UA group |  |  |  |  |  |  |  |  |
| Low | 0.71(0.52-0.97) | 0.031 | 0.73(0.53-0.99) | 0.041 | 0.83 (0.85-1.19) | 0.303 | 0.87 (0.60-1.25) | 0.441 |
| Normal | Reference |  | Reference |  | Reference |  | Reference |  |
| High | 1.30(1.11-1.52) | 0.001 | 1.29(1.01-1.51) | 0.002 | 1.36 (1.14-1.63) | 0.001 | 1.31 (1.09-1.57) | 0.004 |
| Transplant era^a^ | 0.71 (0.56-0.89) | 0.004 | 0.7 (0.55-0.89) | 0.003 | 0.57 (0.45-0.72) | <0.001 | 0.55 (0.43-0.69) | <0.001 |
| Age (years) | 0.98 (0.97-0.98) | <0.001 | 0.98 (0.97-0.98) | <0.001 | 0.98 (0.97-0.99) | <0.001 | 0.98 (0.97-0.99) | <0.001 |
| Sex, male | 0.81 (0.7-0.95) | 0.008 | 0.82 (0.7-0.96) | 0.011 | 0.79 (0.66-0.95) | 0.010 | 0.78 (0.65-0.93) | 0.005 |
| BMI (kg/m^2^) | 1 (0.97-1.02) | 0.904 | 1 (0.97-1.02) | 0.711 | 1.03 (1-1.06) | 0.098 | 1.02 (0.99-1.05) | 0.145 |
| Donor type, deceased | 1.67 (1.19-2.33) | 0.003 | 1.63 (1.17-2.28) | 0.004 | 1.06 (0.67-1.67) | 0.810 | 1.02 (0.65-1.61) | 0.921 |
| Donor age (years) | 1.02 (1.02-1.03) | <0.001 | 1.02 (1.02-1.03) | <0.001 | 1.02 (1.01-1.03) | <0.001 | 1.02 (1.01-1.03) | <0.001 |
| Donor sex, male | 0.99 (0.85-1.14) | 0.860 | 0.99 (0.86-1.15) | 0.930 | 1.01 (0.85-1.19) | 0.947 | 1.02 (0.86-1.21) | 0.834 |
| Pretransplant DM | 1.47 (1.09-2) | 0.012 | 1.47 (1.08-1.99) | 0.013 | 1.77 (1.21-2.58) | 0.003 | 1.8 (1.23-2.63) | 0.002 |
| Duration of pretransplant dialysis (months) | 1 (0.998-1.003) | 0.788 | 1 (0.998-1.003) | 0.840 | 1.001 (0.998-1.003) | 0.717 | 1.001 (0.998-1.004) | 0.545 |
| Retransplantation | 0.8 (0.6-1.06) | 0.113 | 0.82 (0.62-1.09) | 0.167 | 0.78 (0.56-1.1) | 0.159 | 0.8 (0.57-1.13) | 0.202 |
| Number of HLA mismatch | 1.01 (0.95-1.08) | 0.775 | 1.01 (0.94-1.08) | 0.809 | 0.98 (0.9-1.05) | 0.538 | 0.98 (0.91-1.06) | 0.571 |
| Calcineurin inhibitor (Tacrolimus) | 0.65 (0.52-0.82) | <0.001 | 0.67 (0.53-0.84) | 0.001 | 0.8 (0.6-1.06) | 0.120 | 0.77 (0.57-1.02) | 0.071 |
| Delayed graft function | 1.45 (0.86-2.44) | 0.164 | 1.36 (0.81-2.29) | 0.243 | 1.03 (0.46-2.32) | 0.949 | 1.03 (0.46-2.31) | 0.945 |
| BPAR within 1 year | 1.27 (1.07-1.5) | 0.005 | 1.2 (1.02-1.41) | 0.026 | 1.3 (1.08-1.57) | 0.007 | 1.24 (1.03-1.48) | 0.022 |
| SBP at 1 month  (mmHg) | 0.997 (0.987-1.007) | 0.547 | 0.997 (0.987-1.007) | 0.523 | 0.995 (0.983-1.007) | 0.415 | 0.995 (0.984-1.007) | 0.455 |
| DBP at 1 month  (mmHg) | 1.008 (1.001-1.015) | 0.032 | 1.007 (1-1.014) | 0.054 | 1.005 (0.997-1.014) | 0.227 | 1.005 (0.996-1.014) | 0.254 |
| eGFR at 1month (mg/min/1.73m^2^) | 1.006 (1.002-1.010) | 0.003 |  |  | 1.001 (0.996-1.006) | 0.706 |  |  |
| eGFR at 1 year (mg/min/1.73m^2^) |  |  | 1.005 (0.999-1.01) | 0.081 |  |  | 0.995 (0.988-1.001) | 0.089 |

^a^ : after 2004 for the 1-yr analysis, after 2000 for the 5-yr analysis

UA, uric acid; BMI, body mass index; DM, diabetes mellitus; HLA, human leukocyte antigen; BPAR, biopsy-proven acute rejection; SBP, systolic blood pressure; DBP, diastolic blood pressure; eGFR, estimated glomerular filtration rate; HR hazard ratio; CI, confidence interval
